# Computational Approach to Dendritic Spine Taxonomy and Shape Transition Analysis

**DOI:** 10.3389/fncom.2016.00140

**Published:** 2016-12-23

**Authors:** Grzegorz Bokota, Marta Magnowska, Tomasz Kuśmierczyk, Michał Łukasik, Matylda Roszkowska, Dariusz Plewczynski

**Affiliations:** ^1^Centre of New Technologies, University of WarsawWarsaw, Poland; ^2^Nencki Institute of Experimental Biology, Polish Academy of SciencesWarsaw, Poland; ^3^Department of Computer and Information Science, Norwegian University of Science and TechnologyTrondheim, Norway; ^4^Department of Computer Science, University of SheffieldSheffield, UK; ^5^Faculty of Pharmacy, Medical University of WarsawWarsaw, Poland

**Keywords:** dendritic spines, shape transitions, synaptic plasticity, image processing

## Abstract

The common approach in morphological analysis of dendritic spines of mammalian neuronal cells is to categorize spines into subpopulations based on whether they are stubby, mushroom, thin, or filopodia shaped. The corresponding cellular models of synaptic plasticity, long-term potentiation, and long-term depression associate the synaptic strength with either spine enlargement or spine shrinkage. Although a variety of automatic spine segmentation and feature extraction methods were developed recently, no approaches allowing for an automatic and unbiased distinction between dendritic spine subpopulations and detailed computational models of spine behavior exist. We propose an automatic and statistically based method for the unsupervised construction of spine shape taxonomy based on arbitrary features. The taxonomy is then utilized in the newly introduced computational model of behavior, which relies on transitions between shapes. Models of different populations are compared using supplied bootstrap-based statistical tests. We compared two populations of spines at two time points. The first population was stimulated with long-term potentiation, and the other in the resting state was used as a control. The comparison of shape transition characteristics allowed us to identify the differences between population behaviors. Although some extreme changes were observed in the stimulated population, statistically significant differences were found only when whole models were compared. The source code of our software is freely available for non-commercial use^1^. Contact: d.plewczynski@cent.uw.edu.pl.

## 1. Introduction

Brain plasticity depends on the functional and structural reorganization of the synapses. The majority of the excitatory synapses are located on dendritic spines, which are small membranous protrusions localized on the surface of neuronal dendrites. The important feature of dendritic spines is their structural variability, which ranges from long, filopodia spines to short stubby and mushroom-shaped spines. Dendritic spines are typically composed of a head that is connected to the dendrite by a neck. The size of the spine head is proportional to the postsynaptic density area and correlates with the postsynaptic receptor content and synaptic strength (Nusser et al., 1998; Kharazia and Weinberg, 1999; Takumi et al., 1999). The length of the dendritic spine neck is correlated with the postsynaptic potential (Araya et al., 2006; Tønnesen et al., 2014). Thus, the dendritic spine shape has been accepted for determining the strength of the synaptic connections and is thought to underlie the processes of information coding and memory storage in the brain. Furthermore, alterations in dendritic spine shape, size, and density are associated with a number of brain disorders (DeKosky and Scheff, 1990; Irwin et al., 2001; Selemon et al., 2006; Knobloch and Mansuy, 2008; Sweet et al., 2008; Hutsler and Zhang, 2010; Penzes et al., 2011; Levenga and Willemsen, 2012).

The morphology of spines can change in an activity-dependent manner. The structural plasticity of dendritic spines is related to synaptic function, as the morphological modifications of pre-existing spines as well as the formation or loss of synapses accompany the learning and memory processes (Xu et al., 2009; Yang et al., 2009; for reviews see Holtmaat and Svoboda, 2009; Caroni et al., 2012). The cellular models of synaptic plasticity, *long-term potentiation* (LTP) and *long-term depression* (LTD) associate the synaptic strength with spine enlargement and spine shrinkage, respectively (Yuste and Bonhoeffer, 2001; Holtmaat and Svoboda, 2009; Kasai et al., 2010).

Understanding dendritic spine shape taxonomy and shape transitions upon synaptic potentiation is of great importance. The common approach in analysis of dendritic spine morphological changes is to categorize the spines into subpopulations based on whether they are stubby, mushroom, thin, or filopodia shaped (Su et al., 2014). Of importance, the existing categorization of dendritic spine shapes (Su et al., 2014) does not provide a clear definition of each group. Moreover, the literature lacks methods allowing an automated assigment between dendritic spine shapes. Additionally, a recent report suggests the existence of dendritic spine shapes continuum rather than specific spine subclasses (Loewenstein et al., 2015). Thus, the existing classifications have to be revisited and a new automatic classification method with clear mathematical rules should be derived. To address this issue, we employed a new methodological approach with potential applicability in the studies of dendritic spine shape taxonomy and transitions in time. Our clustering-based approach permits analysis of dendritic spine dynamics in multi-dimensional feature space by reducing the complexity subpopulations. To test this method, similarly to previous works, we potentiated the synapses with cLTP stimulation that produces a long-lasting increase in network activity and mimics several aspects of LTP, including synaptic receptor incorporation to the dendritic spine membrane. The morphology of single dendritic spines was assessed using time-lapse imaging of living neurons. In the rest of the paper, we refer to a population of spines stimulated by cLTP as *ACTIVE*, and the non-treated spines are denoted as *CONTROL*.

The paper is organized as follows. In Section 2, we describe the process of data gathering and data representation and the statistical approach to analysis of spine shapes. First, we analyze the basic characteristics of features in the populations named *ACTIVE* and *CONTROL* and conclude that before a meaningful comparison can be performed, the populations need to be normalized. In Section 3, we develop a simple but meaningful numerical representations of spines and we provide an approach to dendritic spine taxonomy construction and models of shape transitions together with statistical tests for model comparisons. For taxonomy development, we propose a clustering-based approach that does not depend on subjective decisions of experts and can accommodate arbitrary numerical features. Later, we introduce a corresponding probabilistic model of spine transitions between the clusters in time. We also propose a bootstrap-based approach and two statistical tests that are applied for the purpose of the comparison of models built for different populations of spines. Finally, in Section 4, we present an example of method application. We summarize our work in Section 5.

## 2. Data preparation and analysis

In this section, we describe the statistical analyses of mammalian dendritic cell populations *ACTIVE* and *CONTROL*. A comparison of the descriptor distributions showed that an initial data preprocessing was necessary, which we performed by carefully choosing subsets of the spines from both populations^2^. Finally, we introduce the algorithm for spine representation dimensionality reduction.

### 2.1. Data acquisition

Dissociated hippocampal cultures were prepared as described previously in Nunez (2008). On the 10th day, *in vitro* cells were transfected using Effectene (Qiagen) according to the manufacturer's protocol with a plasmid carrying red fluorescence protein under β-actin promoter. All the experiments were performed over the course of 19–21 days *in vitro*. During the imaging session, the cells were kept in an acquisition chamber with controlled temperature (37°C) and stable CO_2_ (5%) concentration. Chosen dendritic segments decorated with dendritic spines were imaged at the two time points: at the time 0 (before the stimulation) and 10 min after the stimulation. In the ACTIVE group the chemical LTP (cLTP) was induced by bath application of a mixture of 50 μM forskolin, 50 μM picrotoxin and 0.1 μM rolipram (each dissolved in DMSO) in maintenance media. The CONTROL group received compound-free solvent (DMSO). Of note, the implemented way of stimulation (cLTP) mimics several LTP aspects such as enhanced network activity of the hippocampal neurons (Niedringhaus et al., 2012), delivery of the extrasynaptic AMPA-receptors (Oh et al., 2006), and changes in the dendritic spine structure (Szepesi et al., 2014).

Image acquisition was performed using the Leica TCS SP 5 confocal microscope with a PL Apo 40 × /1.25 NA oil immersion objective using a 561 *nm* line of diode pumped solid state laser with 10% transmission and collected date with a pixel size of 1024 × 1024. Captured cell images consisted of series of z-stacks taken at every 0.4μ*m* step. On average, around 14–17 slices (depending on specimen thickness) were taken per stack. The final sampling density was 0.07μ*m* per pixel.

The resolution of the confocal microscope along the optical axis (z axis) is three time worse than the resolution along the lateral direction. The majority of observed dendritic spines arise in the lateral direction. Thus, due to limitations of confocal microscopy, it is almost impossible to determine the three-dimensional dendritic spine features. The spines that could be easily distinguished and that protruded in the transverse direction were chosen for analysis. Because of the synaptic scaling, dendritic spine structure and density are modulated with respect to the position along the dendritic tree (Menon et al., 2013). To avoid this issue and following the approach by Michaluk et al. (2011), we chose spines that belonged to the secondary dendrites.

The next step of data preparation was to obtain the numerical features of the spines. Although many spine extraction methods exist (Fanti et al., 2011; Schmitz et al., 2011; Li and Deng, 2012), the methods do not prove to be useful in our approach. Therefore, we analyzed the images semi-automatically using custom written software (Ruszczycki et al., 2012). The recorded dendritic spine features (denoted as *DESCRIPTORS*) were length, head width (denote hw), max width location (denote mwl), max width (denote mw), neck width (denote nw), foot, circumference, area, width to length ratio (denote wlr), length to width ratio (denote lwr), and length to area ratio (denote lar). Since in this paper we focus on the two dimensional data, the direct dendritic spine volume analysis is not possible. However, the volume can be estimated based on the area. Moreover, the method *per se* can be used to analyze the three dimensional data. Although researches have not found a consensus yet on which features should be considered, this set covers parameters that are the most often used (Michaluk et al., 2011; Szepesi et al., 2014; Tønnesen et al., 2014). The spine length was determined by measuring the curvilinear length along the virtual skeleton of the spine, which was obtained by fitting the curve (using fourth-degree polynomial). The fitting procedure involved searching for a curve along which the integrated fluorescence was at a maximum level. Many spines were distinctly bent such that the distance along a straight line between the tip and the base of the spine underestimates the length of the spine. To define the head width, we used the diameter of the largest spine section that was perpendicular to the virtual skeleton, while the bottom part of the spine (third of the spine length adjacent to the dendrite) was excluded. To define the neck width, we used the thinnest part of the spine between the position of the head-width measurement and the point at which the spine is anchored into the dendrite. The details can be found at work done by Ruszczycki et al. (2012).

We ended up with two groups of spines, the stimulated *ACTIVE* type consisting of 433 samples and the control *CONTROL* type consisting of 490 samples. For each spine, all of the above 11 features were measured at two different timestamps: *t*_0_ (the time before stimulation) and *t*_1_ (10 min after *t*_0_). It has been shown that that after 10 min (Szepesi et al., 2014), modifications in the spine structure could already be observed and demonstrated that stimulation causes the cleavage of important adhesion molecules at the dendritic spines (Stawarski et al., 2014). Consequently, by *ACTIVE* (*CONTROL*), we denote all features at all timestamps and, by *ACTIVE*^*x*^, we denote all spines from the *ACTIVE* data set described only by features at time *t*_*x*_ (similarly, *CONTROL*^*x*^).

### 2.2. Balanced subset selection

In Table 1, we report the mean values for descriptors from *ACTIVE*^0^ and *CONTROL*^0^ populations. We report *p*-values from two-tailed *t*-tests^3^ for the difference of means between both sets. We report significant differences for almost all descriptors (only for three features is the *p*-value above the threshold value *p*>0.001).

**Table 1:** **Differences between ***ACTIVE*** and ***CONTROL*** at time ***t***_**0**_**.

**Feature**	******ACTIVE******^**0**^		******CONTROL******^**0**^		***p*-value**
	**Mean**	**Std**	**Mean**	**Std**
Length	1.268	0.470	1.539	0.714	0.000
Head width	0.685	0.098	0.808	0.092	0.000
Max width location	0.554	0.083	0.608	0.073	0.003
Max width	0.792	0.134	0.958	0.108	0.000
Width length ratio	0.667	0.168	0.657	0.142	0.721
Length width ratio	2.161	2.391	2.223	3.390	0.577
Neck width	0.418	0.081	0.551	0.090	0.000
Foot	0.772	0.133	0.994	0.219	0.000
Circumference	4.612	3.969	5.502	6.223	0.000
Area	0.675	0.183	0.977	0.307	0.000
Length area ratio	2.158	0.966	1.726	0.597	0.000

*Means and p-values from the two-tailed t-test. Width length ratio and length width ratio are dimensionless, area is in μm^2^ and length area ratio is in μm^−1^. Other descriptor values are measured in μm. Significant differences are observed between all descriptor values except for two features*.

Such large differences between both sets may influence the statistical analysis of their behavior. Therefore, we decided to preprocess the datasets by excluding some spines, such that the means in the new sets are similar with respect to the statistical test used. Namely, we drew a number of pairs of closest spines, each pair consisting of a spine from the *ACTIVE* set and a spine from the *CONTROL*. The measure of how close the spines are is based on the normalized Euclidean distance^4^ between the vectors of features at time *t*_0_. The pseudo-code for the algorithm is presented in Algorithm S1 in the Supplementary Material.

Such an approach can be justified by the fact that the two cultures were obtained from different animals (each culture consist of a mix of dissociated hippocampal neurons isolated from one litter of rat pups), thus a systematic differences might appear between *in vitro* cultures. Initially dendritic spines may exhibit a significant structural differences between cultures, yet here we focus on the direction of stimulation-induced structural changes. The implemented standardization allowed us to eliminate the changes unrelated to the treatment and highlight the stimulation-related modifications in dendritic spine structure between the analyzed groups^5^. One can not exclude a possibility that the change in the dendritic spines may be dependent on their initial morphological features (Kasai et al., 2003; Szepesi et al., 2014). Thus, the implemented normalization of the dendritic spine shape at the beginning of the analysis should not affect the obtained outcome.

In Table 2, we report new statistics on the differences between samples after the 300 closest pairs have been drawn^6^. The same statistical test that was performed before is used here as well. The *p*-values are significantly higher for all features, and no one feature is significantly different in the two compared groups. We are going to further investigate these new “normalized” sets, denoted as *ACTIVE300* (the 300 closest spines drawn from *ACTIVE*) and *CONTROL300* (the 300 closest spines drawn from *CONTROL*).

**Table 2:** **Differences between ***ACTIVE300***^0^ and ***CONTROL300***^**0**^ at time ***t***_**0**_**.

**Feature**	******ACTIVE******^**0**^		******CONTROL******^**0**^		***p*-value**
	**Mean**	**Std**	**Mean**	**Std**
Length	1.240	0.286	1.276	0.288	0.416
Head width	0.736	0.070	0.743	0.064	0.762
Max width location	0.592	0.075	0.586	0.071	0.789
Max width	0.844	0.076	0.845	0.062	0.977
Width length ratio	0.702	0.137	0.688	0.132	0.630
Length width ratio	1.898	1.251	1.917	1.231	0.832
Neck width	0.479	0.075	0.505	0.069	0.236
Foot	0.840	0.122	0.855	0.127	0.606
Circumference	4.566	2.329	4.591	2.136	0.834
Area	0.720	0.126	0.748	0.121	0.330
Length area ratio	1.871	0.498	1.837	0.492	0.556

*Means and p-values from two-tailed t-tests are shown. Width length ratio and length width ratio are dimensionless, area is in μm^2^ and length area ratio is in μm^−1^. Other descriptor values are measured in μm. No significant differences between any descriptor values are observed for all geometrical features*.

## 3. Methods

In this section, we present PCA and apply two clustering methods to construct the spine shape taxonomy in an unsupervised way. Further, we build the probabilistic model of shape changes in time. Finally, the bootstrap analysis is presented to statistically evaluate the differences between both the resting and potentiated populations.

### 3.1. Simplification of shape representations

The Principal Component Analysis (PCA) (for details see Jolliffe, 2002) is used where the reduction of the data dimensionality is required. It has been used in order to provide the most significant information. The PCA method provides orthogonal basis, which is required for Euclidian metrics used for clustering. We applied PCA to spines from both populations *CONTROL* and *ACTIVE* and for both *t*_0_ and *t*_1_. For the first two features (components) in the reduced representation, we cover about 91% of the variance in the data (see Figure S1). The removal of other features does not reduce the available information by much (only 9% of the variance is lost). The new features are linear combinations of the initial features: *Comp*.1′ = −0.27 · *length* − 0.49 · *lwr* − 0.81 · *circumference* − 0.15 · *area*; *Comp*.2′ = − 0.17 · *hw* − 0.17 · *mw* − 0.11 · *wlr* + 0.71 · *lwr* − 0.12 · *nw* − 0.12 · *foot* − 0.41 · *circumference* − 0.21 · *area* + 0.44 · *lar*. We see that *Comp*.1′ is composed mostly of features related to size such as length, circumference, and area. Therefore, this feature can be treated as a generalized size descriptor. Similarly, we can interpret *Comp*.2′ as a generalized contour (shape slenderness) descriptor.

The interpretation of the above components as size and contour descriptors allows us to construct more meaningful features. The initial features can be directly divided into two sets: *DESCRIPTORS*^*SIZE*^ ={length, circumference, area} (size related features) and *DESCRIPTORS*^*CONTOUR*^ ={hw, foot, mwl, mw, wlr, lwr, lar, nw} (contour related features). Then, PCA is applied separately to each of the sets. Using the first feature from PCA on *DESCRIPTORS*^*SIZE*^ and the first feature from PCA on *DESCRIPTORS*^*CONTOUR*^, 87% of the variance is explained (see Figure S2). The loss of the variance compared with PCA computed on all features merged together is equal to 4%. However, the new representation (denoted as *DESCRIPTORS*^*PCA*^) is easy to interpret. The new features provide a clear meaning of size and contour slenderness and reduce to a simple form:

$$\begin{array}{l} \begin{array}{c} Comp.1=-0.29\cdot length-0.94\cdot circumference-0.19\cdot \;area\\ Comp.2=-0.14\cdot wlr+0.94\cdot lwr+0.28\cdot \;lar \end{array} \end{array}$$

Comparing the loadings (weights) against previous formulas for *Comp*.1′ and *Comp*.2′, we notice that the differences are small, i.e., below 15% in most cases. The most important feature of the size descriptor is the circumference (the highest loading), and the most important feature of the contour descriptor is lwr. Most of the initial features, i.e., hw, foot, mwl, mw, and nw, are not included (We used the Principal Component Analysis function (*princomp*) which is implemented in programming language *R*. Feature weights smaller than 10% were neglected.

**Figure 1:**
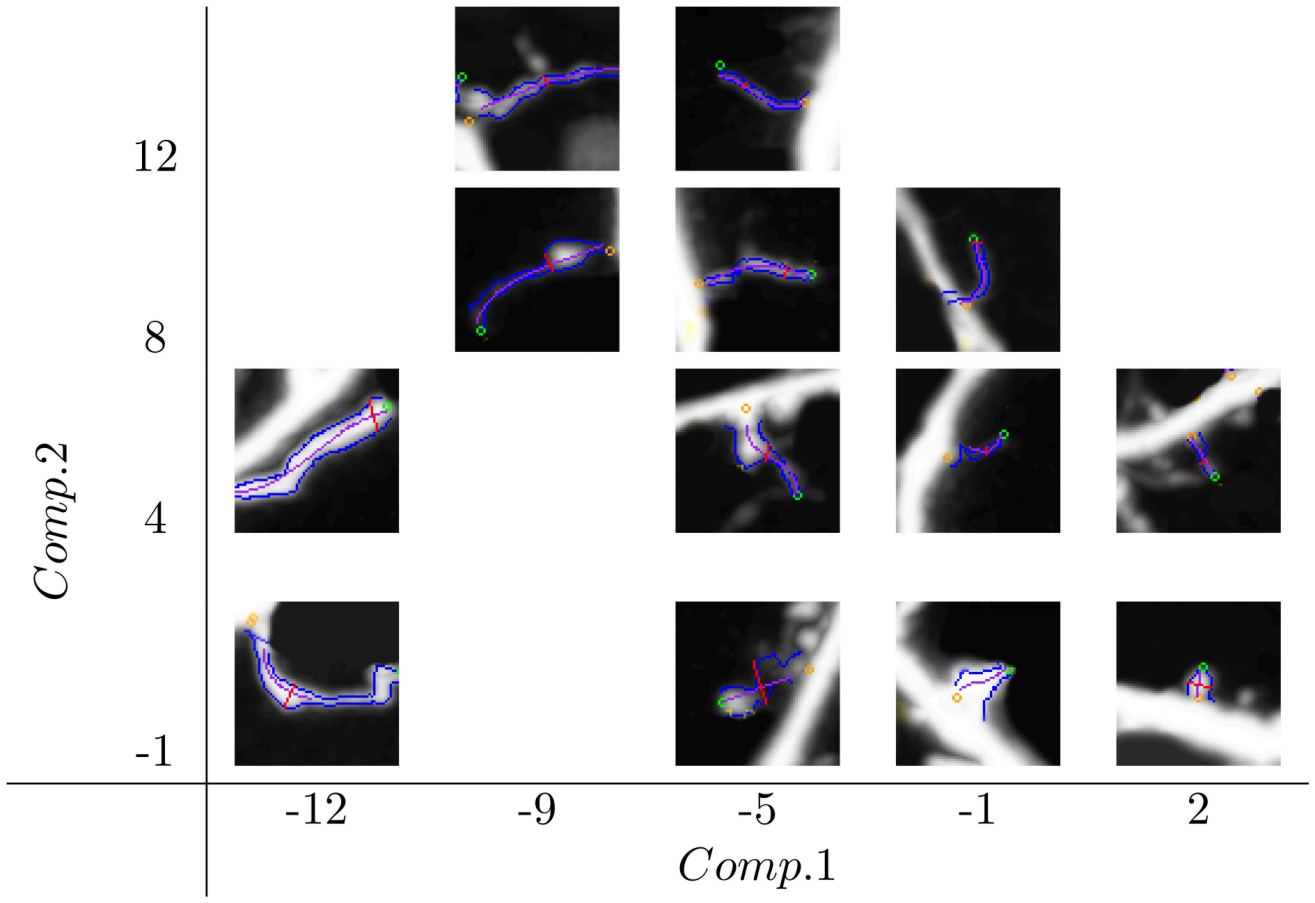
**Distribution of spine shapes in space composed of the features ***Comp***.1 and ***Comp***.2**. *Comp*.1 is a generalized size descriptor, and *Comp*.2 is a generalized spine slenderness. Spine sizes change along *Comp*.1 from the smallest on the right side to the biggest on the left side. The spine contour slenderness changes along *Comp*.2 from the thinnest on the top to the thickest on the bottom.

Spine distributions in the new feature space *Comp*.1 × *Comp*.2 are shown in Figure 1. The whole space of features was partitioned into tiles of size 4 × 4, and for each tile, one representative spine (the closest to the tile center) was chosen. We can see how the spine size changes along *Comp*.1 from the smallest on the right side to the biggest one on the left side. Similarly, spine slenderness change along *Comp*.2, from the thinnest on the top to the thickest on the bottom.

### 3.2. Clusters of shapes

Initially, the spines are represented in some arbitrary multidimensional space of features, e.g., *DESCRIPTORS*^*PCA*^. Our goal is to obtain a high-level representation that would be both meaningful and simple. Therefore, we propose to apply clustering. Clustering allows for assigning similar objects in terms of their geometrical properties (for example, spines) to groups; i.e., subsets that we call clusters in our paper. In this study the clusters represent the possible shapes of spines. The underlying idea is that the spines in a cluster have greater similarity shapes (they are more similar in terms of derived features) among themselves than to spines outside the given cluster. We consider two well-established algorithms, *cmeans* (Bezdek, 1981) and average-linkage *hierarchical* (Murtagh, 1983), that represent the two main types of clustering: *crisp* and *fuzzy*.

In clustering, each spine *s* is assigned a vector *w*(*s*) = (*w*_1_(*s*), …, *w*_*k*_(*s*)) of *k* membership weights that are non-negative and sum up to 1. For example, *w*_*n*_(*s*) is a membership of the spine *s* against the *n*-th cluster. In *crisp* clustering, spines are assigned to exactly one cluster (*w*_*n*_(*s*) = 1 ⇔ *s* assigned to n-th cluster; 0 otherwise). In *fuzzy* clustering, weights can be arbitrary real numbers between 0 and 1. Additionally, weights can be interpreted as probabilities, e.g., *w*_*n*_(*s*) can be interpreted as the probability that spine *s* belongs to the *n*-th cluster.

To obtain a taxonomy of shapes that would describe the spines in both time points equally well, we applied clustering to data *ACTIVE* ∪ *CONTROL* from both time points *t*_0_ and *t*_1_. Consequently, each spine was included twice and assigned two vectors of weights. Spine *s* at time *t*_0_ is assigned the vector *w*^0^(*s*) and at time *t*_1_ the vector *w*^1^(*s*). We denote $w_{n}^{i}\left(s\right)=P^{i}\left(s\in C_{n}\right)$ as the probability that spine *s* belongs to cluster *n* at time *t*_*i*_.

The above clustering algorithms have either one (*hierarchical*) or two (*cmeans*) parameters: *k* - number of clusters and *m* - fuzzifier (indicates cluster fuzziness). A large *m* results in smaller weights and more fuzzy clusters. For small *m*, e.g., *m* = 1, we obtain results close to *crisp* clustering. Consequently, low values of both *k* and *m* are preferred. Although these parameters can be selected in many ways, we decided to use the *Within Cluster Sum of Squares* (*WSS*) (Thorndike, 1953). The definition of *WSS* is as follows: $WSS=\underset{n=1..k}{\sum }\underset{s}{\sum }w_{n}\left(s\right){\left(\vec{s}-\vec{c_{n}}\right)}^{2}$ where $\vec{c_{n}}=\frac{\underset{s}{\sum }w_{n}\left(s\right)\cdot \vec{s}}{\underset{s}{\sum }w_{n}\left(s\right)}$ is the *n*-th cluster centroid and $\vec{s}$ stands for the vector of features assigned to object *s*. *WSS* has several good properties; i.e., simple meaning, applicability to both crisp and fuzzy cases, and the same behavior no matter what data and what clustering algorithm are used (it decreases when *k* increases and when *m* decreases). For a balance between the number of clusters, data fitness values of *k* and *m* at the “knee point” (the point where *WSS* plot bends the most) should be selected.

### 3.3. Shape transition model

#### 3.3.1. Assumptions and brief description

It has been showed that the initial dendritic spine morphology may influence how this structure will change upon specific treatment (Matsuzaki et al., 2004; Szepesi et al., 2014); i.e., the induction of long-term potentiation. Therefore, we assume that changes of spines depend on their initial shapes and that each spine follows patterns of behavior highly correlated with its initial shape. We introduce a novel probabilistic model of behavior that relies on these principles.

The shapes of dendritic spines are represented as weights of shape clusters *w*_*n*_(*s*). The changes of spines shapes are represented by the combinations of behavior patterns represented with probabilities *P*(*C*_*n*_ → *C*_*m*_|*C*_*n*_) or the probability that the shape represented by cluster *C*_*n*_ will change into the shape represented by cluster *C*_*m*_ when *t*_0_ → *t*_1_. Probabilities *P* can be stored in a *k*×*k* matrix called *transition matrix*, where rows are enumerated with *n* and columns with *m*. An even more convenient representation of the same information is a graph, where nodes represent shape clusters and edges are labeled with probabilities, denoted as a *transition graph* (i.e., **Figures 3, 4**).

#### 3.3.2. Probability estimation

In the *crisp*, e.g., *hierarchical* model of shapes, we can estimate the probability *P* as follows: $P_{crisp}\left(C_{n}\to C_{m}|C_{n}\right)=\frac{\underset{s}{\sum }w_{n}^{0}\left(s\right)\cdot w_{m}^{1}\left(s\right)}{\underset{s}{\sum }w_{n}^{0}\left(s\right)}$. In the denominator, we have the number of spines that belong to cluster *C*_*n*_ in time *t*_0_ (normalizer). In the nominator, there is the number of spines that belong to cluster *C*_*n*_ in time *t*_0_ and to cluster *C*_*m*_ in time *t*_1_ (recall that only for one *n* in $w_{n}^{0}\left(s\right)$ and for one *m* in a $w_{m}^{1}\left(s\right)$, the values are ones; elsewhere, they are zeros). With such a computation, we consider how many spines moved from shape cluster *C*_*n*_ to *C*_*m*_ and normalize it by the number of all spines in the initial cluster *C*_*n*_.

There are arbitrarily many generalizations that are consistent with the above crisp derivation for the fuzzy model, (e.g., the *cmeans* model); i.e., $w_{n}^{0}\left(s\right)\cdot w_{m}^{1}\left(s\right)$ can be reformulated in many ways without changing the values of *P*_*crisp*_, e.g., as $\min\left(w_{n}^{0}\left(s\right),w_{m}^{1}\left(s\right)\right)$. We suggest using the generalization for which the model minimizes the prediction error for the distribution of shapes at time *t*_1_. The probability that spine *s* in time *t*_1_ will be in cluster *C*_*m*_ for our linear model is given according to the law of total probability as follows:

(1) $$\begin{array}{l} P_{prediction}^{1}\left(s\in C_{m}\right)=\underset{n}{\sum^{\text{​}}}P\left(C_{n}\to C_{m}|C_{n}\right)\cdot P^{0}\left(s\in C_{n}\right) \end{array}$$

The overall prediction error can be computed as a sum of squared differences between predicted ($P_{prediction}^{1}$) and derived probabilities (*P*^1^):

(2) $$\begin{array}{l} E=\underset{s}{\sum^{\text{​}}}\underset{m}{\sum^{\text{​}}}{\left(P_{prediction}^{1}(s\in C_{m})-P^{1}(s\in C_{m})\right)}^{2} \end{array}$$

where for each spine *s* in the data, we compare the membership for cluster *C*_*m*_ at time *t*_1_ with the prediction of the model. The problem can be now formulated as an optimization task where we search for probabilities *P*(*C*_*n*_ → *C*_*m*_|*C*_*n*_) that minimize the overall prediction error *E*:

(3) $$\begin{array}{l} objective\;:\;argminE\\ subject\;to\;:\\ \;P\left(C_{n}\to C_{m}|C_{n}\right)\geq 0\\ \;\forall_{s}\forall_{n}{\sum^{\text{​}}}_{m}P\left(C_{n}\to C_{m}|C_{n}\right)=1 \end{array}$$

The above derivations can be easily represented in matrix form, and the above optimization problem is an example of a standard quadratic programming optimization task with constraints. The details are presented in Section S2 in Supplementary Material.

#### 3.3.3. Prediction of the weights at time t1

To verify the model (for example, in the case of cross-validation procedure), or apply it to new datasets, we implemented the computational method to estimate structural changes (described here as 2D descriptors) of a spine in time. The estimation of weights in time *t*_1_ for a new spine *s* (not in the training data) is not always obvious. For *hierarchical* clustering, we used a first nearest neighbor classifier for parameters in time *t*_0_; i.e., we searched for the most similar sample vector (*s*′), in time *t*_0_, from the training data and assigned *w*(*s*) = *w*(*s*′), where *w*^0^(*s*) is the classification and *w*^1^(*s*) is the estimation of changes of *s* in time. For *cmeans* clustering, the prediction of weights of a new spine (*s*) is more straightforward. Each spine, whether from the training data or not, has weights (*w*^0^) assigned according to the same explicit formula: $w_{n}^{0}\left(s\right)=\frac{v_{n}\left(s\right)}{\sum_{i=1}^{k}v_{i}\left(s\right)}$ where $v_{n}\left(s\right)=\frac{1}{\sum_{j=1}^{k}{\left(\frac{\left||s-{\text{c}}_{n}|\right|}{\left||s-{\text{c}}_{j}|\right|}\right)}^{\frac{2}{m-1}}}$ where **c**_*n*_ is the *n*-th cluster centroid. This calculates the probability that a given spine is in the *n*-th cluster based on the distance from the cluster centroid. Estimation of the weights *w*^1^(*s*) can be calculated as matrix-multiplication with transition matrix *w*^1^(*s*) = *Pw*^0^(*s*), where *P* = [*p*_*i, j*_] and *p*_*i, j*_ = *P*(*C*_*j*_ → *C*_*i*_|*C*_*j*_).

#### 3.3.4. Parameter reliability

To derive information on the reliability of the obtained probabilities, we use the following bootstrap-based procedure. We generate *R* = 1000 new populations sampled with replacement from the original population. For each new population, we calculate all the probabilities again. The average squared differences between the probabilities for the new populations and the original populations are used as the estimates of parameter errors. Formally, the error of the probability *P*(*C*_*n*_ → *C*_*m*_|*C*_*n*_) is calculated as:

(4) $$\begin{array}{l} SE_{nm}=\sqrt{\frac{1}{R}\underset{r=1..R}{\sum^{\text{​}}}\left(P(C_{n}\to C_{m}|C_{n}\right)-P{\left(C_{n}\to C_{m}|C_{n})|r\right)}^{2}} \end{array}$$

where *P*(*C*_*n*_ → *C*_*m*_|*C*_*n*_)|*r* denotes the probability calculated for the *r*-th bootstrap population.

### 3.4. Comparison of models

Bootstrap Hypothesis Testing (Efron and Tibshirani, 1993) is a method of testing statistical hypotheses. To apply the method, one has to first modify the testing sample so that the null hypothesis is satisfied. Subsequently, a large number of bootstrap samples are drawn from such a modified sample. Finally, for the fixed statistic of interest, one must evaluate how extreme the value of the statistic is for the original sample compared with the values obtained for the drawn bootstrap samples.

This general rule in our case proceeds as follows. We take the two groups *ACTIVE300* and *CONTROL300* and join them into one group *ACTIVE300* ∪ *CONTROL300*. This group contains information about changes between t0 and t1 time. At each iteration of bootstrap sampling, two new groups are drawn from the joint dataset. This way, the null hypothesis of a common distribution for both groups is satisfied. Next, for each bootstrap, the sample clusters and *Shape Transition Model* are constructed for both groups. Then, the test statistic is computed. Finally, the statistic are computed on models built from the original groups and compared with the bootstrap sampling results.

#### 3.4.1. Comparison of changes in cluster distributions

We cluster spines according to their shapes (see Section 3.2). As a result, for each spine *s* at *t*_0_ and *t*_1_, we obtain the set of weights representing a mixture of shapes. Then, we derive the overall distribution (total weights) of shapes (by shapes, we mean clusters of shapes) at both *t*_0_ and *t*_1_. The *n*-th cluster total weight (in the case of *crisp*; e.g., *hierarchical* clustering, it is equivalent to the number of spines) in *t*_0_ is equal to $\sum_{s}w_{n}^{0}\left(s\right)$ and in *t*_1_ is equal to $\sum_{s}w_{n}^{1}\left(s\right)$. Consequently, the relative change in the *n*-th cluster weight between *t*_0_ and *t*_1_ for population *G* can be computed as follows: $c_{n}\left(G\right)=\frac{\sum_{s\in G}w_{n}^{1}\left(s\right)-\sum_{s\in G}w_{n}^{0}\left(s\right)}{\sum_{s\in G}w_{n}^{0}\left(s\right)}$. The statistic that measures the difference between relative changes in distributions of shapes for populations *G*_1_, *G*_2_ can be now defined as:

(5) $$\begin{array}{l} RDC(G_{1},G_{2})=\underset{n=1..k}{\sum^{\text{​}}}{\left(c_{n}(G_{1})-c_{n}(G_{2})\right)}^{2} \end{array}$$

#### 3.4.2. Comparison of transition matrices

By applying the *Shape Transition Model* (see Section 3.3), we construct two Markov matrices (*transition matrices*) describing the transitions for both populations. To check how similar the matrices are, we also decided to apply bootstrap hypothesis testing. For comparing the matrices, we use the sum of squared differences between corresponding cells from the two matrices:

(6) $$\begin{array}{l} SMD(G_{1},G_{2})=\underset{n=1..k}{\sum^{\text{​}}}\underset{m=1..k}{\sum^{\text{​}}}{\left(P_{nm}|G_{1}-P_{nm}|G_{2}\right)}^{2} \end{array}$$

where *G*_1_, *G*_2_ are populations, e.g., *ACTIVE300*, *CONTROL300*, to be compared. *P*_*nm*_|*G*_*i*_ ≡ *P*(*C*_*n*_ → *C*_*m*_|*C*_*n*_)|*G*_*i*_ stands for the value of a cell in the *n*-th row and in the *m*-th column of the transition matrix *P* built with data from population *G*_*i*_.

## 4. Results

Here, we provide an example of an application of the method. To obtain the taxonomy of spine shapes, we applied *cmeans* and *hierarchical* clustering to *DESCRIPTORS*^*PCA*^ for *ACTIVE* ∪ *CONTROL* for *t*_0_ and *t*_1_. To select the proper values of the parameters, we used *WSS* plots with “knee” shapes (see Figure S3). We obtained *k* = 10 for *hierarchical* and *k* = 8, *m* = 4 for *cmeans* clustering. According to *WSS* measures, these values ensure a good balance between the complexity of the results; i.e., the number of clusters and quality of cluster fitness.

**Figure 2:**
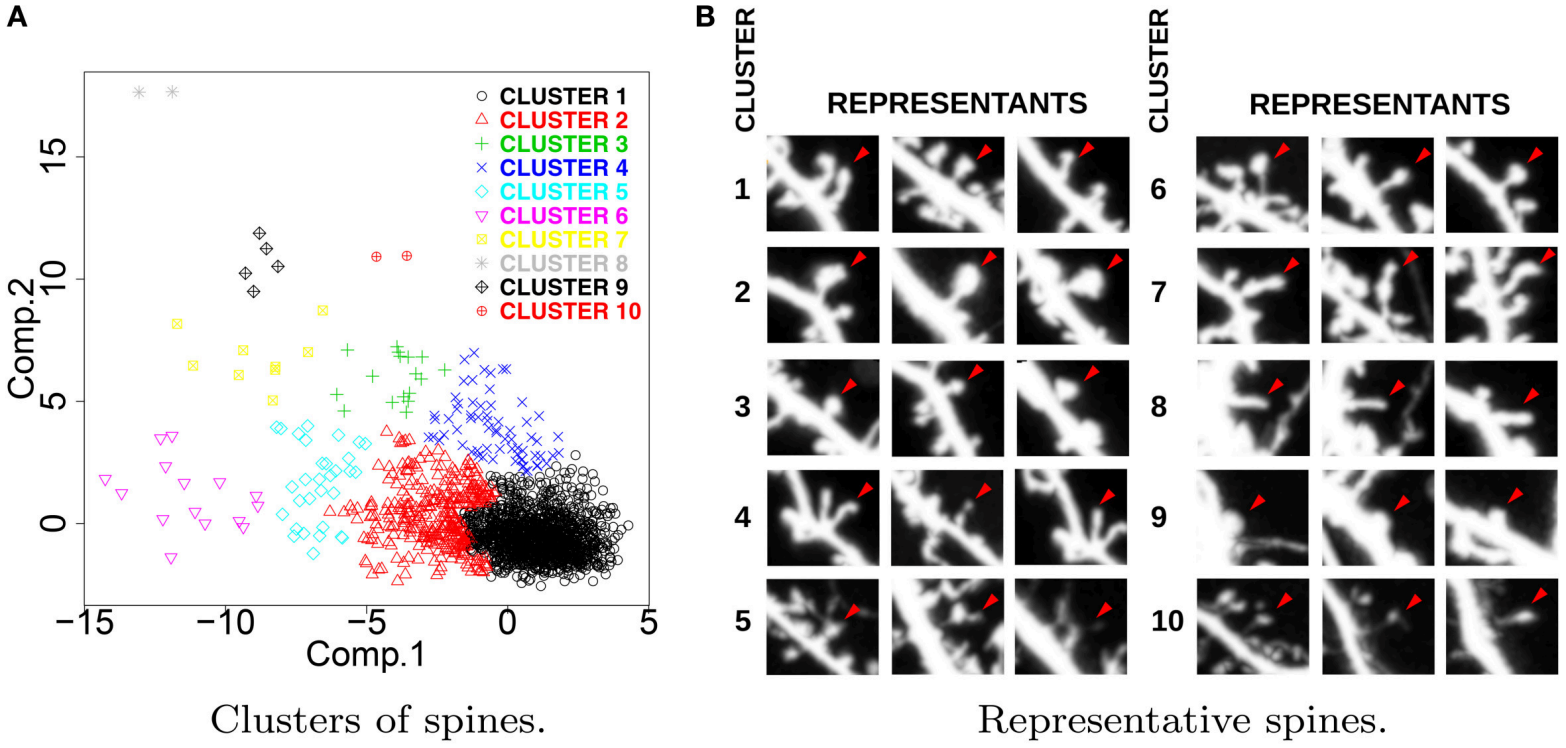
**(A)** Distribution of spine clusters obtained by *hierarchical* clustering. **(B)** Representative spines obtained for *ACTIVE* ∪ *CONTROL*. The presented clusters represent the universal taxonomy of spine shapes. For each cluster, we present three spines that are nearest to the cluster center. Representative spines facilitate visual aid for interpretation purposes.

Figure 2A presents the results of *hierarchical* clustering calculated for *ACTIVE* ∪ *CONTROL* according to the procedure described in Section 3.2. Each spine is represented with a single point, and the colors represent the cluster memberships. For each cluster, we identified three representative spines lying nearest to the cluster center. Representative spines are shown in Figure 2B. The obtained clusters express the universal taxonomy of shapes that will be later employed for the computation of *Shape Transition Model* for *ACTIVE*, *CONTROL*, *ACTIVE300*, and *CONTROL300*. Representative spines can be used for visual inspection and biological interpretation.

Apart from *hierarchical* clustering, we also consider *cmeans* clustering. Table 3 presents the comparison of the prediction error *E* for both methods. Values were obtained using 10-fold cross-validation. For the purposes of cross-validation, the dataset was randomly separated into ten (10) subsets. Numbers from the same column but in different rows should not be compared. Different clustering methods result in different shape clusters that have different members and thus are incomparable. Although the errors *E* for different methods have different ranges and cannot be compared, different models with the same method can be compared.

**Table 3:** **Prediction error ***E*** for various models and clustering methods**.

**Clustering method**	***Shape transition model***	**Majority vote**	**No transitions**	**Random transitions**
*hierarchical*	0.266 ± 0.147	0.395 ± 0.237	0.433 ± 0.242	0.997 ± 0.124
*cmeans*	0.024 ± 0.004	0.853 ± 0.029	0.037 ± 0.007	0.054 ± 0.012

*Values were obtained using 10-fold cross-validation on ACTIVE∪CONTROL. Values in columns should not be compared. For both clustering methods, Shape Transition Model performs better than the baseline*.

The *Shape Transition Model* is compared with three baselines. The first baseline is the majority vote model, where all spines from a particular cluster move to a single destination cluster that is selected as the most popular choice. The second baseline is the model, where we assume that all spines remain in the initial clusters, i.e., weights in *t*_1_ are the same as in *t*_0_. Finally, the third baseline assumes random values for the probability *P*. For both clustering methods, the *Shape Transition Model* has the smallest error *E* and predicts spine behavior the best.

**Figure 3:**
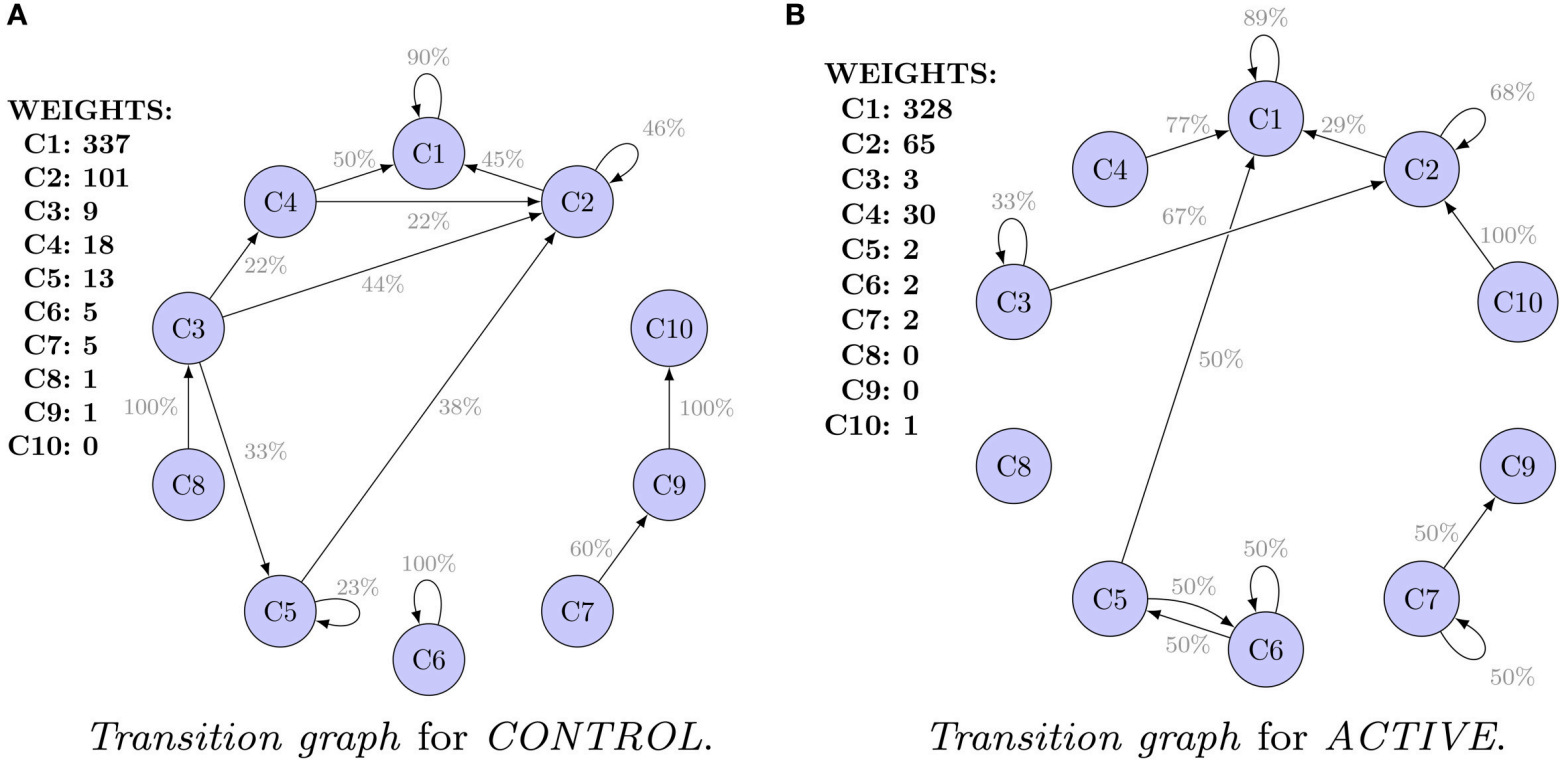
*****Transition graphs*** for ***hierarchical*** clustering**. For each cluster, the initial weight (number of spines in the cluster) is presented. Only transitions (probabilities) of values higher than 20% are shown. Only clusters 1–5 are well represented in the data. Transitions for the remaining clusters are uncertain. **(A)** *Transition graph* for *CONTROL*. **(B)** *Transition graph* for *ACTIVE*.

*Transition graphs* of the *Shape Transition Model* for *ACTIVE* and *CONTROL* are shown in Figures 3A,B. Each cluster of shapes is represented by an oval. Initial sizes, i.e., weights of clusters (for *hierarchical* clustering equivalent to number of spines), are listed. Edges representing transitions are labeled with probability *P*. They are filtered out, and only transitions (probabilities) greater than 20% of the initial weight are visible.

Only five clusters (numbers 1–5) are well represented in the data. Clusters 1, 2, and 4 are the most dense. Clusters 3 and 5 are interpreted as peripheral. Finally, clusters 6–10 have only a few spines. For transitions from clusters 6–10, high errors were obtained. For example, *SE* for *P*(*C*_9_ → *C*_10_|*C*_9_) is equal to 66%. Conclusions concerning clusters 6–10 are not reliable. Analogous plots of clustering results and *transition graphs* for *cmeans* are presented in Figures S4, S5A,B.

**Figure 4:**
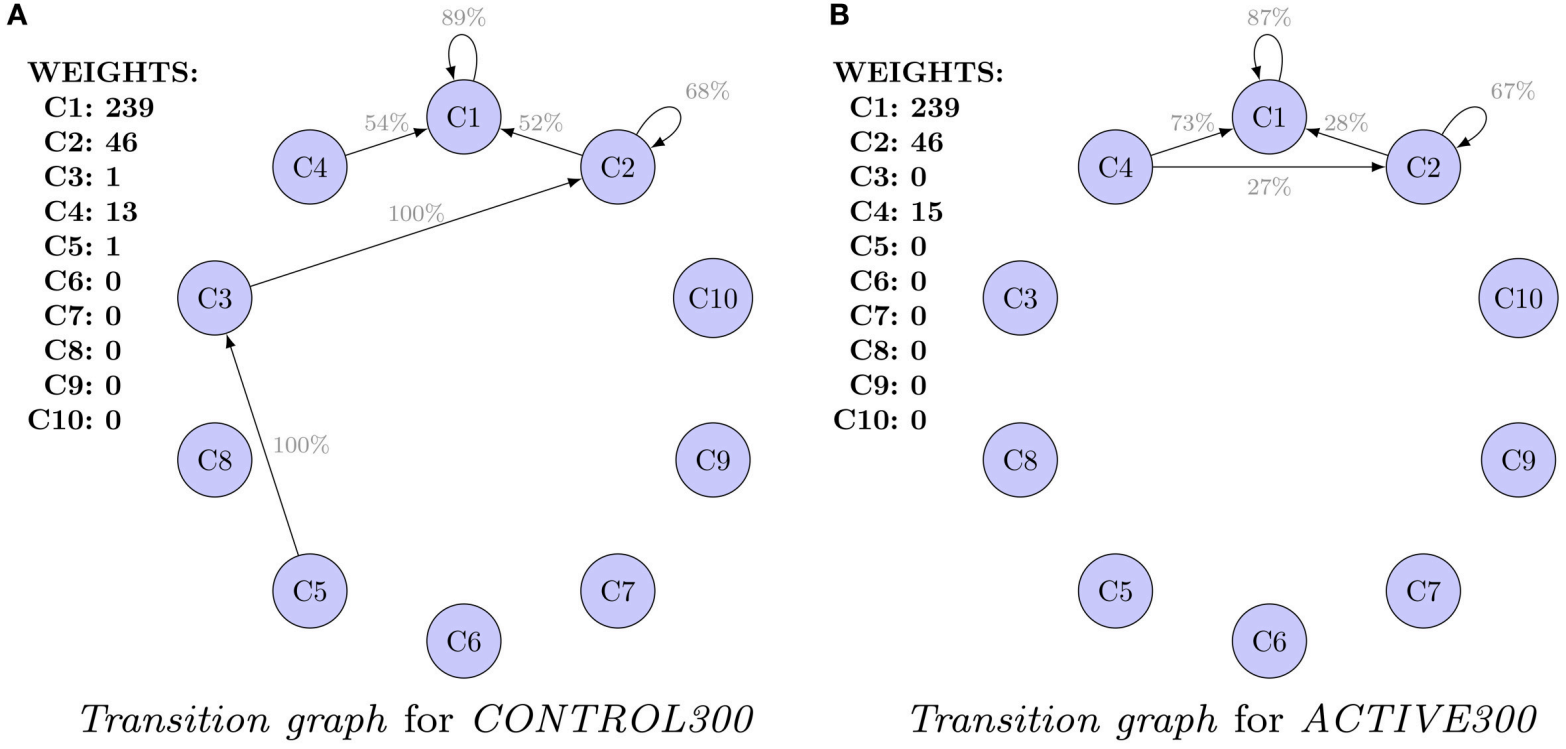
**Comparison of the ***transition graphs*** for balanced subpopulations and ***hierarchical*** clustering**. For each cluster, the initial weight (number of spines in the cluster) is presented. Values are given in percents. Only transitions (probabilities) of values higher than 20% are shown. Differences in transitions between graphs are observed, but because of high uncertainties, none of them is significant. **(A)** *Transition graph* for *CONTROL300*. **(B)** *Transition graph* for *ACTIVE300*.

Graphs presented in Figures 3A,B should not be compared because they are computed for populations of different characteristic at *t*_0_. Alternatively, Figure 4 presents a comparison of the *transition graphs* for *CONTROL300* and *ACTIVE300* for *hierarchical* clustering (the exact values of the probabilities can be found in Table S1). A similar analysis for *cmeans* is presented in Figure S6, and the values of the transitions in percent can be found in Table S2.

In the case of the *CONTROL300* and *ACTIVE300* subsets (Figure 4), only clusters 1, 2, and 4 contain enough spines to produce credible conclusions. For *CONTROL300*, cluster 1 has a slightly stronger inertia than for *ACTIVE300* (91 vs. 87% spines remained in the same cluster). For cluster 2, the situation is the opposite: 41% of spines from cluster 2 for *CONTROL300* remain in cluster 2 compared with 67% for *ACTIVE300*. For both populations, a large transition of spines from cluster 2 to cluster 1 is observable. However, for *CONTROL300*, it is present for 52% of the spines, whereas for *ACTIVE300*, it is present only for 28%. Another difference is visible for transitions from cluster 4. For *CONTROL300*, 73% of spines move to cluster 1 and 27% to cluster 2. For *ACTIVE300*, only 54% of spines move to cluster 1, and the rest move to clusters 2–5. Unfortunately, none of the observed differences is significant when the errors are taken into consideration. Therefore, to identify such differences, the models must be compared as a whole.

Table 4 presents *p*-values of *RDC* and *SMD* statistics (Equations 5 and 6) used for a comparison of models for *ACTIVE300* and *CONTROL300*. Results below 0.05 are marked in bold font. Detailed plots of the statistical distributions using kernel estimation are shown in Figures S7, S8. For *hierarchical* clustering, only *SMD* shows a significant difference between *ACTIVE300* and *CONTROL300*. This statistic compares transitions of spines between shapes, which is well captured by *hierarchical* clustering. The *RDC* statistics depend only on changes of distributions, and *hierarchical* clustering enforces that each spine belongs to only one shape cluster at the particular time point, which may noticeably affect the overall distributions. In contrast, the distributions are well captured by *cmeans* clustering, where each spine is an arbitrary mixture of shapes and *RDC* shows a significant difference. Different clustering methods are sensitive to different properties of the data. The selection of the right clustering method and appropriate test depends on the characteristic of the data that is of interest to the researcher.

**Table 4:** *****P***-values of ***RDC*** and ***SMD*** statistics with bootstrap tests used to compare balanced subpopulations ***ACTIVE300*** and ***CONTROL300*** for various clustering methods**.

**Method**	***RDC p*-value**	***SMD p*-value**
*hierarchical*	0.493	**0.011**
*cmeans*	**0.004**	0.298

*Differences that are statistically significant are shown in bold font*.

## 5. Discussion and conclusions

The majority of excitatory synapses in the brain are located on dendritic spines. These highly dynamic and plastic structures undergo constant morphological changes in different physiological and pathological processes (Kasai et al., 2010). The structure of the dendritic spines is tightly correlated with their function and reflects the synapse properties. Synapse strengthening or weakening along with dendritic spine formation and elimination assure correct processing and storage of the incoming information in the neuronal network. This plastic nature of the dendritic spines allows them to undergo activity-dependent structural modifications, which are thought to underlie learning and memory formation. At the cellular level, the most extensively studied aspect of this phenomena is related to dendritic spine enlargement in response to stimulation.

In this study, we show a computational method that permits statistical analysis of the impact of an externally applied stimulation on the dendritic spine structural dynamics. We applied statistical tests and examined a population consisting of 923 dendritic spines. We used two dissociated neuronal cell cultures and compared the dendritic spine volume and shape changes between two populations at two different states, unstimulated (*CONTROL*) and LTP-stimulated (*ACTIVE*), and at two time points (with a 10-min time interval). We preprocessed the datasets and reduced the dendritic spine number to 300 for each analyzed group.

We provided a probabilistic model for dendritic spine population dynamics. First, the resting state model was constructed (Figure 3A). Then, the probabilistic null model for active neurons was built (Figure 3B). We showed that LTP treatment induced transition of filopodia-like spines (cluster **4**) into mushroom-shaped spines (cluster **2**). For the first time, we provided exact transition probabilities for this morphological transformation (from cluster **4** to cluster **2**, the transition probability was found to be 0.27 ± 0.11). Our result show that in *ACTIVE300* in comparision to *CONTROL300* there is significant group of growing neurons which partially supports the previous studies (Szepesi et al., 2014) that report chemical LTP-induced spine enlargement in dissociated cultures.

Finally, we compared the models for balanced populations (Figure 4). We found differences between active and non-active neurons. Unfortunately, none of the observed differences between the models was significant when particular transitions between shape clusters were considered. Such absence of significant differences can be explained by too small number of spines in analyzed data. Large errors predominated the differences between values in cells of appropriate transition matrices. However, statistically significant differences were detected when models of populations were compared. Different clustering algorithms showed statistically significant differences between the two analyzed groups (*ACTIVE300, CONTROL300*). Crisp clustering captured the difference in shapes transitions well, whereas fuzzy clustering captured the difference in changes of shape cluster distributions.

## Ethics statement

All experimental procedures were carried out in accordance with the Ethical Committee on Animal Research of the Nencki Institute, based on the Polish Act on Animal Welfare and other national laws that are in full agreement with EU directive on animal experimentation.

## Author contributions

MM and MR performed biological experiments and did manually segmentation of spines from confocal microscope images. TK, MŁ, GB, and DP designed the computational method and performed statistical analysis. GB contributed in the data analysis and visualization. All authors read and approved the final manuscript.

## Funding

TK and MŁ were partially supported by research fellowships within “Information technologies: research and their interdisciplinary applications” agreement POKL.04.01.01-00-051/10-00. MM and MR were supported by the grants no. 6651/B/P01/2011/40 and UMO-2015/17/B/NZ3/00557 from National Science Centre Poland. DP, GB, TK, and MŁ was supported by the Polish National Science Centre (Grant numbers 2013/09/B/NZ2/00121 and 2014/15/B/ST6/05082) and COST BM1405 and BM1408 EU actions.

### Conflict of interest statement

The authors declare that the research was conducted in the absence of any commercial or financial relationships that could be construed as a potential conflict of interest.

## Footnotes

^2^Even under ideal experimental conditions, variation among the spines is still present; thus, we choose to standardize them. This practice allows us to start with more homogeneous data and to reveal subtle differences.

^3^The null hypothesis is that the means are equal, and the alternative is that the means are different.

^4^Each feature is normalized by subtracting the mean and dividing by the standard deviation, both calculated based on the feature values from both the ACTIVE and CONTROL sets.

^5^Systematic differences between the analyzed populations were observed and it is noted that the implemented statistical normalization could cause certain biases.

^6^We tried various different numbers of spines (100, 200, 300, 400) and concluded that 300 is the largest which satisfies the desired condition for assessing similarity between different spines.
