# Supplementary material for: Computational Approach to Dendritic Spine Taxonomy and Shape Transition Analysis
Source: Front Comput Neurosci. 2016 Dec 23;10:140. doi: 10.3389/fncom.2016.00140 (PMC5180374; doi:10.3389/fncom.2016.00140)
Supplement: Supplementary file 1 [file DataSheet1.pdf]

Computational approach to dendritic spine taxonomy and shape transition analysis

Supplemental Materials

Grzegorz Bokota [4], Marta Magnowska [3], Tomasz Kusmierczyk [1], Michal Lukasik [2], Matylda Roszkowska [3], Dariusz Plewczynski [4,5]

[1] Department of Computer and Information Science, Norwegian University of Science and Technology, [2] Department of Computer Science, University of Sheffield, [3] Nencki Institute of Experimental Biology, Polish Academy of Sciences, [4] Centre of New Technologies, University of Warsaw, Poland, [5] Faculty of Pharmacy, Medical University of Warsaw, Poland

S1 Algorithms for subset selection

Large differences between the sets *ACTIVE* and *CONTROL* may influence the statistical analysis of their behavior. Therefore, we decided to preprocess the datasets by excluding some spines, such that the means in the new sets are close with respect to the statistical test used. Below we show the pseudocode for algorithm, where the subsets of spines are selected, forming new sets for further analysis.

Algorithm S1 SUBSET-SELECTION

Input:
 Lists of spines: *ACTIVE* and *CONTROL*
 A function of state of all variables: *STOP CONDITION*
Output:
 Lists of spines: *ACTIVESUBSET* and *CONTROLSUBSET*

1: Normalize each feature of *ACTIVE* and *CONTROL* by subtracting the common mean and dividing by the common standard deviation,
2: Initialize *ACTIVESUBSET* and *CONTROLSUBSET* to empty lists,
3: **while** *STOP CONDITION* is not satisfied **do**
4:     draw the pair of spines $x1 \in ACTIVE$ and $x2 \in CONTROL$ of the smallest euclidean distance
5:     move $x1$ and $x2$ from their lists respectively to *ACTIVESUBSET* and *CONTROLSUBSET*
6: **end while**
7: **return** *ACTIVESUBSET* and *CONTROLSUBSET*

S2 Matrix formulation of Shape Transition Model

Shape Transition Model can be represented in the matrix form where:

- W^i - $N \times k$ matrix of weights where each row represents a single spine at time t_i
- P - $k \times k$ matrix of transition probabilities $P(C_n \rightarrow C_m | C_n)$ indexed by n and m.

Predictions of the model can be calculated as follows:

$$W^1_{prediction} = W^0 P$$

Prediction error can be calculated as follows ($||A|| \equiv \sqrt{\sum_{ij} A_{ij}^2}$):

$$E = ||W^1_{prediction} - W^1||^2$$

The optimization problem is given by: (1_k - k-element vertical vector of ones):

$$\begin{aligned}
&objective : argmin_P \ ||W^0 P - W^1||^2 \\
&subject\ to : \\
&\ P \geq 0 \\
&\ P \cdot 1_k = 1_k
\end{aligned}$$

and can be transformed to the standard quadratic programming form:

$$\begin{aligned}
&objective : argmin_x \ \tfrac{1}{2} x^T Q x + c^T x \\
&subject\ to : \\
&\ A \cdot x \leq b \\
&\ Aeq \cdot x = beq
\end{aligned}$$

For this we use that:

$$\begin{aligned}
\|W^0 P - W^1\|^2 &= \sum_{i,j} ((W^0 P)_{i,j} - W^1_{i,j})^2 \\
&= \underbrace{\sum_{i,j} ((W^0 P)_{i,j})^2}_{\frac{1}{2} x^T Q x} \underbrace{- \sum_{i,j} 2 (W^0 P)_{i,j} W^1_{i,j}}_{c^T x} + \underbrace{\sum_{i,j} (W^1_{i,j})^2}_{const}
\end{aligned}$$

such that we arrive at the following parameters for the quadratic programming problem:

- $x = flatt(P)$ is a vector of length k^2 where, for a square matrix of size $k \times k$:

$$flatt(P) = \left[P_{l \bmod k, \lfloor \frac{l}{k} \rfloor}\right]_{l=0}^{k^2}$$

- $Q = [q_{i,j}]$ where

$$q_{i,j} = \begin{cases} \sum_{l=0}^{N} W_{l,i} W_{l,j} & \lfloor \frac{i}{k} \rfloor = \lfloor \frac{j}{k} \rfloor \\ 0 & otherwise \end{cases}$$

- $c = -flatt([W_{i,j}^0 W_{i,j}^1]_{i,j})$ is a vector of length k^2
- $A = -I$ where I is an identity matrix of size $k^2 \times k^2$
- $b = 0_{k^2}$ is a vector of zeros of length k^2
- $Aeq = [a_{i,j}]$ where

$$a_{i,j} = \begin{cases} 1 & \lfloor \frac{i}{k} \rfloor = \lfloor \frac{j}{k} \rfloor \\ 0 & otherwise \end{cases}$$

- $beq = 1_{k^2}$ is a vector of ones of length k^2

This quadratic programming problem can be solved for example with the *quadprog* R package.

S3 Supplemental figures and tables

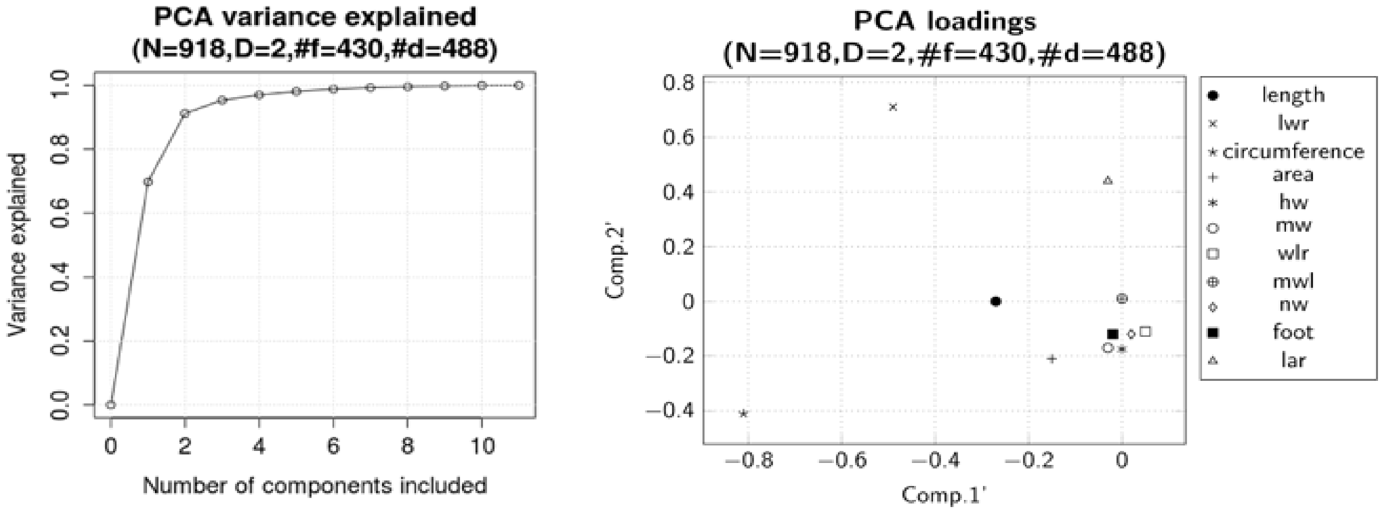

Fig. S1 Proportion of the explained variance for different numbers of components (left) and loadings (weights) for two of the most important components (right). PCA was calculated on $DESCRIPTORS$ of $CONTROL \cup ACTIVE$ data. For two features (components) about 91% of the variance is explained. We see that $Comp.1'$ is composed mostly of features related to size such as length, circumference, and area. Therefore, this feature can be treated as a generalized size descriptor. Similarly, we can interpret $Comp.2'$ as a generalized contour (shape slenderness) descriptor. N=number of spine, D=number of dimensions, #f number of spines from ACTIVE group, #d number of spines from CONTROL group

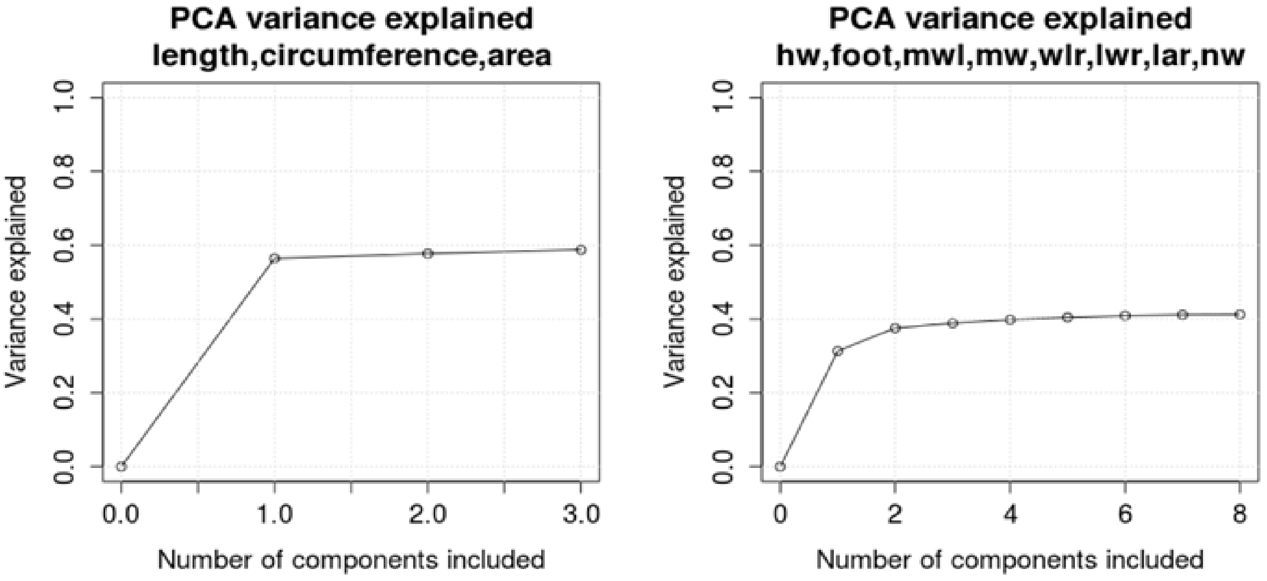

Fig. S2 Proportion of the explained variance for PCA on components (features) describing size (left) and contour (right). PCA was calculated separately on $DESCRIPTORS^{SIZE} =$ {length, circumference, area} (size related features) and on $DESCRIPTORS^{CONTOUR} =$ {hw, foot, mwl, mw, wlr, lwr, lar, nw} (contour slenderness related features) of $CONTROL \cup ACTIVE$ data. Using the first feature from PCA on $DESCRIPTORS^{SIZE}$ and the first feature from PCA on $DESCRIPTORS^{CONTOUR}$ 87% of the variance is explained.

Table S1 *Transition matrices* $t_0 \rightarrow t_1$ for *CONTROL300* and *ACTIVE300* for *hierarchical* clustering. Values are denoted in percents, *SE* in brackets, source clusters in rows, and destination clusters in columns. Only clusters 1, 2 and 4 contain enough spines to produce credible conclusions. According to estimated errors, transitions observed for other cases are not meaningful.

CONTROL300

From \ To	1	2	3	4	5	6	7-10
1	91 (34)	6 (2)	0	2 (1)	0	0	0
2	52 (20)	41 (16)	0	2 (1)	2 (1)	2 (1)	0
3	0	100 (58)	0	0	0	0	0
4	54 (22)	15 (8)	8 (5)	15 (8)	8 (5)	0	0
5	0	0	100 (63)	0	0	0	0
6	0	0	0	0	0	0	0
7-10	0	0	0	0	0	0	0

ACTIVE300

From \ To	1	2	3	4	5	6	7-10
1	87 (32)	12 (4)	0	0	0	0	0
2	28 (11)	67 (25)	0	2 (1)	2 (1)	0	0
3	0	0	0	0	0	0	0
4	73 (29)	27 (11)	0	0	0	0	0
5	0	0	0	0	0	0	0
6	0	0	0	0	0	0	0
7-10	0	0	0	0	0	0	0

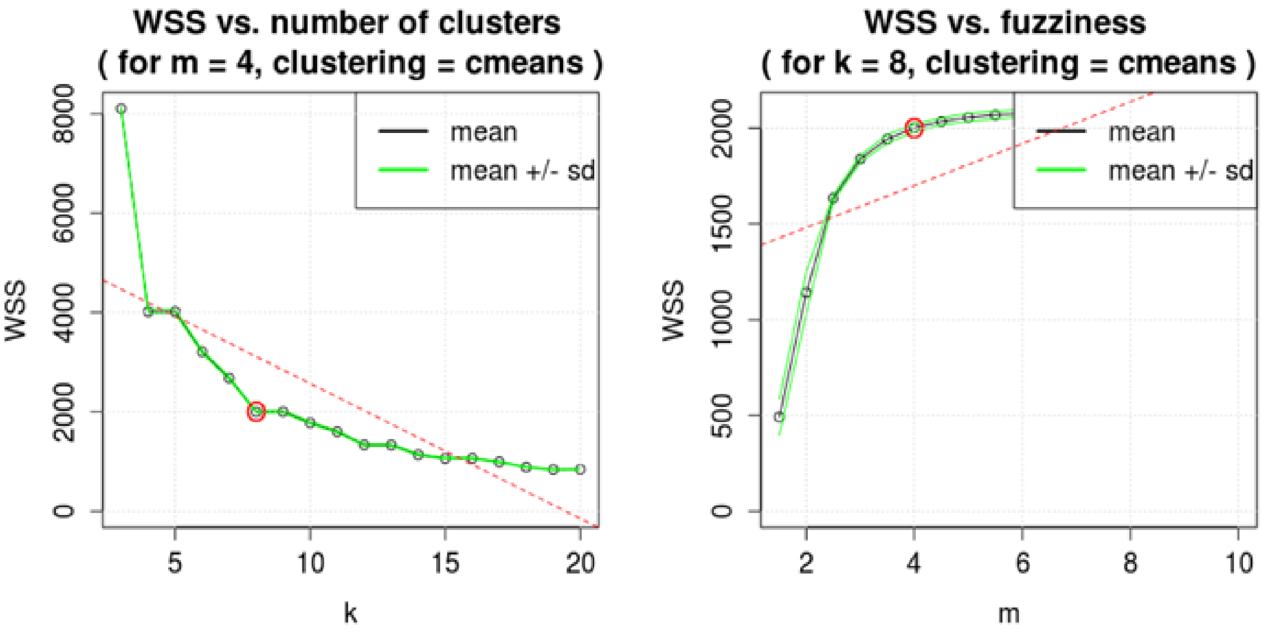

(a) *cmeans* ($k = 8$, $m = 4$)

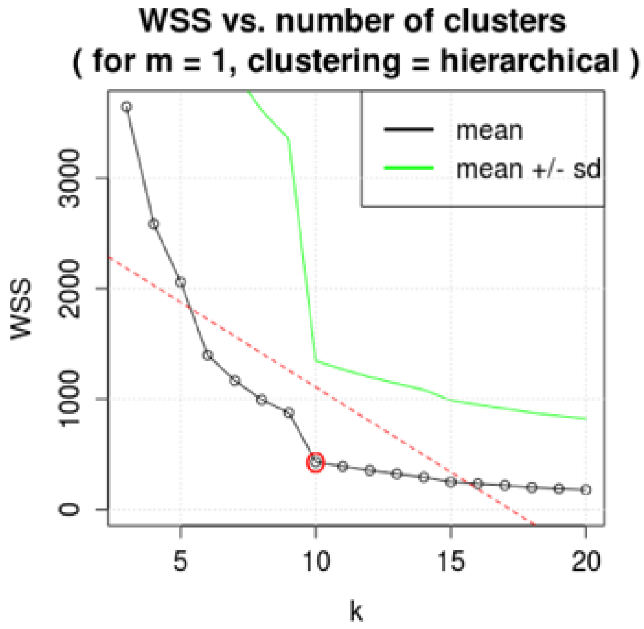

(b) *hierarchical clustering* ($k = 10$)

Fig. S3 Parameters selection for shape clustering methods (WSS plots for $ACTIVE \cup CONTROL$). Red circles mark the selected values in 'knee points'. The dashed line is the result of linear regression. The knee point is the most distant point on the WSS plot measured from linear regression and localized between two intersections of WSS and linear regression curves.

Table S2 *Transition matrices* $t_0 \rightarrow t_1$ for *CONTROL300* and *ACTIVE300* for *cmeans* clustering. Values are denoted in percents, *SE* in brackets, source clusters in rows, and destination clusters in columns. In contrast to *hierarchical* clustering case, all clusters contain nonneglible amounts of spines.

CONTROL300

From \ To	1	2	3	4	5	6	7	8
1	43 (18)	5 (4)	0 (1)	4 (3)	22 (11)	0 (4)	0 (3)	25 (12)
2	2 (1)	17 (6)	13 (5)	10 (4)	3 (2)	21 (8)	28 (10)	6 (3)
3	0 (0)	5 (2)	50 (19)	0 (1)	0 (0)	15 (6)	29 (12)	1 (1)
4	1 (1)	19 (7)	5 (2)	35 (13)	10 (4)	14 (7)	4 (2)	12 (5)
5	11 (5)	11 (5)	3 (2)	23 (9)	27 (12)	3 (3)	2 (2)	20 (8)
6	2 (1)	20 (8)	10 (4)	8 (4)	5 (3)	40 (14)	15 (6)	0 (1)
7	5 (2)	15 (6)	19 (8)	17 (7)	4 (2)	10 (4)	21 (8)	9 (4)
8	4 (3)	19 (8)	2 (2)	14 (6)	17 (8)	9 (5)	11 (6)	24 (10)

ACTIVE300

From \ To	1	2	3	4	5	6	7	8
1	33 (15)	19 (9)	7 (4)	0 (4)	11 (7)	0 (1)	12 (6)	19 (9)
2	2 (1)	23 (9)	5 (3)	25 (10)	5 (3)	15 (8)	8 (4)	16 (7)
3	1 (1)	13 (5)	40 (15)	2 (1)	0 (0)	13 (5)	27 (10)	3 (1)
4	0 (0)	14 (6)	14 (6)	20 (9)	4 (2)	31 (13)	15 (6)	2 (2)
5	19 (9)	0 (1)	0 (0)	21 (10)	46 (18)	0 (0)	0 (1)	14 (6)
6	5 (2)	13 (5)	21 (8)	5 (3)	3 (1)	30 (12)	19 (7)	6 (3)
7	2 (1)	15 (5)	16 (6)	16 (6)	4 (2)	18 (7)	24 (9)	6 (2)
8	9 (4)	15 (6)	1 (1)	19 (7)	18 (8)	0 (0)	5 (2)	33 (13)

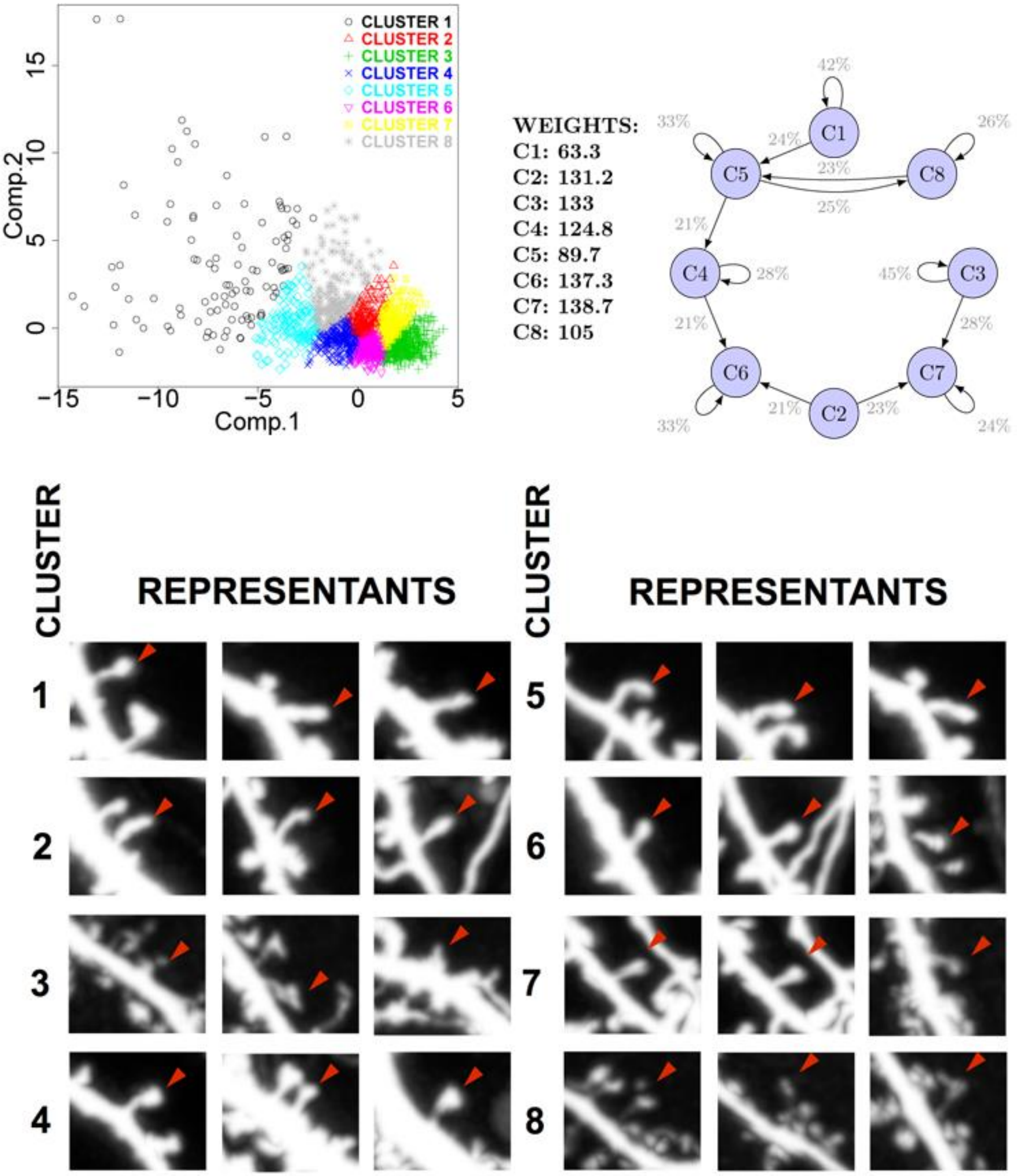

Fig. S4 Results (clusters plot, *transition graph* and spines selected as clusters' representants) of *cmeans* clustering applied to $ACTIVE \cup CONTROL$. For each cluster, the initial weight (sum of weights of spines in the cluster at time t_0) is presented. Only transitions of values higher than 20% are shown on the graph. In contrast to *hierarchical* clustering case, all clusters contain nonneglible amounts of spines. However, differences between spines from different clusters are not that significant and easy to interpret.

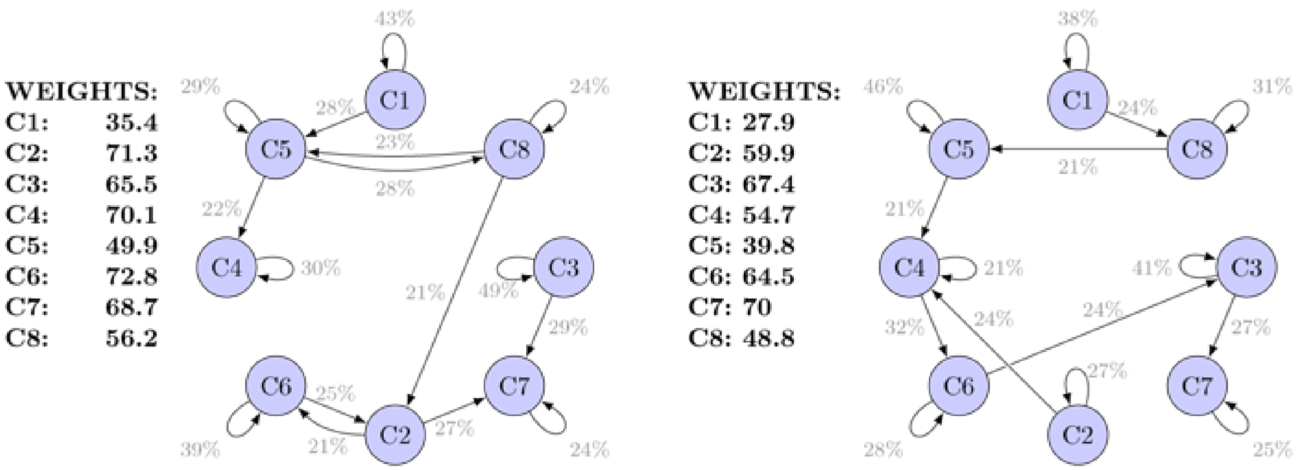

(a) *CONTROL* (b) *ACTIVE*

Fig. S5 *Transition graphs* for for *cmeans* clustering. For each cluster the initial weight (sum of spines' weights in the cluster at time t_0) is presented. The transition probabilities between clusters (indicated in rounded-of percent) are noted at the respective edges of the transition graph. The percentages are calculated for each cluster separately. Only transitions (probabilities) of values higher than 20% are shown. Subfigures should not be compared because they are computed for populations of different characteristic at t_0.

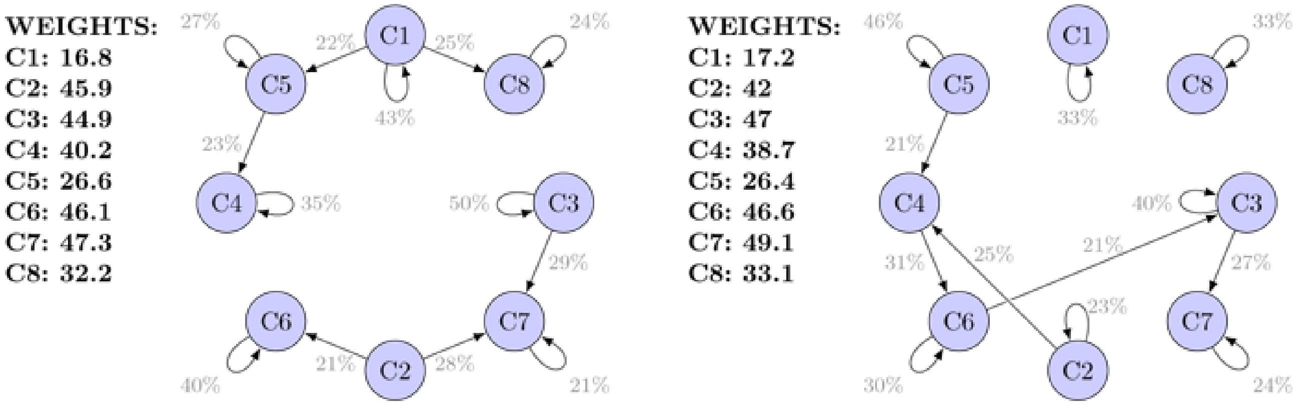

(a) *CONTROL300* (b) *ACTIVE300*

Fig. S6 *Transition graphs* for balanced subpopulations and *cmeans* clustering. For each cluster, the initial weight (sum of spines' weights in the cluster at time t_0) is presented. The transition probabilities between clusters (indicated in rounded-of percent) are noted at the respective edges of the transition graph. The percentages are calculated for each cluster separately. Only transitions of values higher than 20% are shown.

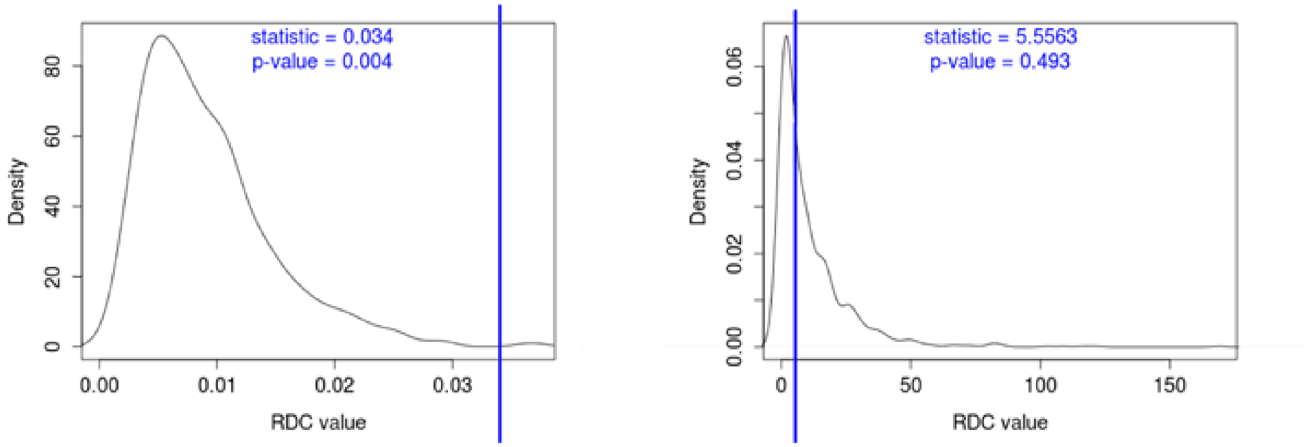

(a) *RDC* distribution for *cmeans* clustering

(b) *RDC* distribution for *hierarchical* clustering

Fig. S7 The probability density plots obtained from a bootstrap for *RDC* statistic used to compare *CONTROL300* and *ACTIVE300*. Kernel estimation used for smoothing. Statistically significant difference between subpopulations were observed for *cmeans* case.

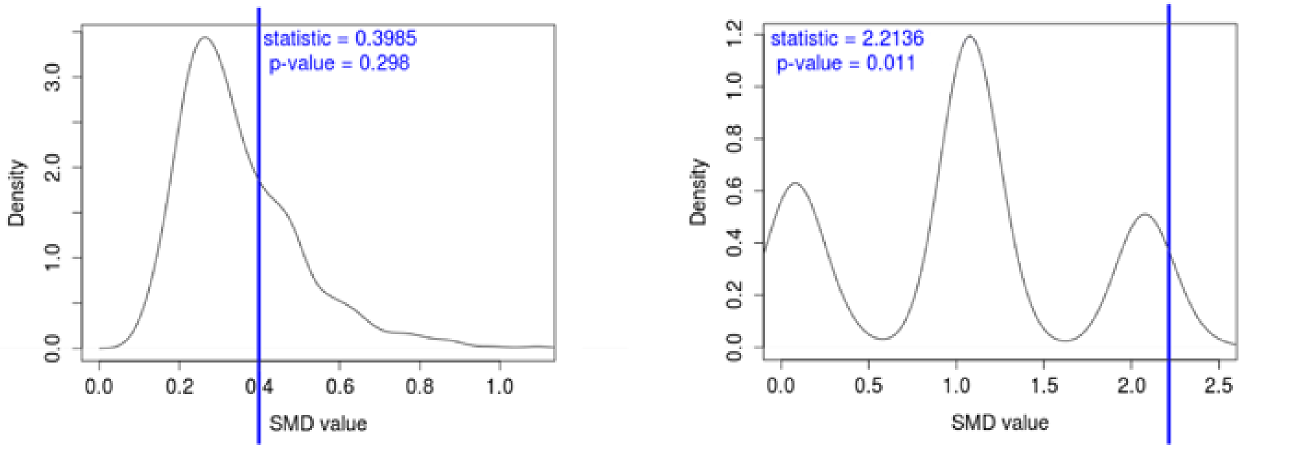

(a) *SMD* distribution for *cmeans* clustering

(b) *SMD* distribution for *hierarchical* clustering

Fig. S8 The probability density plots obtained from a bootstrap for *SMD* statistic used to compare *CONTROL300* and *ACTIVE300*. Kernel estimation used for smoothing. Statistically significant difference between subpopulations were observed for *hierarchical* case.
